# Gender disparities in healthy ageing in China: current status and future prospects

**DOI:** 10.3389/fpubh.2025.1587922

**Published:** 2025-05-21

**Authors:** Jiajia Deng, Lingshuai Kong, Wenyu Li

**Affiliations:** ^1^School of Economics and Management, Changji University, Xinjiang, China; ^2^College of Military and Political Basie Education, National University of Defense Technology, Changsha, China; ^3^School of Marxism, Capital Normal University, Beijing, China

**Keywords:** population aging, gender disparities, healthy aging, gender-sensitive strategies, aging

## Abstract

As the global population ages, China faces unique challenges due to its rapid economic development and societal changes. The older adult population in China, especially females, is growing rapidly, with women outnumbering men in older age groups. Gender disparities in aging manifest in physiological, psychological, and social aspects, including higher risks of cardiovascular diseases in older adultmen and osteoporosis in older adult women. China’s rapidly aging population faces profound gender disparities in health outcomes, shaped by biological, social, and cultural determinants. Synthesizing data from the Global Burden of Disease study and national surveys, this perspective highlights elevated cardiovascular risks among older adult men, osteoporosis prevalence in women, and systemic inequities in healthcare access. We propose gender-sensitive strategies spanning research, policy, and societal awareness to advance equitable healthy aging.

## 1 Introduction

Population aging has emerged as a non-negligible social phenomenon with the change of global population. According to a United Nations survey,^1^ the number of older adult individuals (aged 65 and above) has doubled, from approximately 260 million in 1980 to 761 million in 2021. Between 2021 and 2050, the global proportion of the older adult population is expected to increase from below 10% to approximately 17%.

Population aging not only signifies a continuous rise in the proportion of the older adult but also represents a series of challenges that society must confront, including safeguarding the health and quality of life of the older adult (1). Particularly in China, with rapid economic development and profound societal changes, the issue of population aging has become increasingly salient. Data from the Seventh National Population Census in China indicate^2^ that individuals aged 60 and above currently account for 18.70% of the total population, with those aged 65 and above comprising 13.50%, signifying a significant acceleration in the aging process. From 2010 to 2020, the proportion of individuals aged 60 and above increased by 5.44 percentage points, while those aged 65 and above rose by 4.63 percentage points. Compared to the previous decade, these increments were higher by 2.51 and 2.72 percentage points, respectively, indicating that China has entered an aging society prematurely. Against this backdrop, healthy aging has become a critical issue. In its 2015 Global Report on Ageing and Health, the WHO defined healthy aging as “optimizing opportunities for physical, social, and mental health to enable older people to take an active part in society and enjoy life.” Emphasizing health and vitality in the physical, psychological, and social dimensions of the older adult, healthy aging serves as a key strategy to address the challenges of an aging society. Globally, countries are actively exploring ways to achieve healthy aging, and as the country with the largest older adult population in the world, China faces unprecedented challenges and opportunities (2).

However, a frequently overlooked perspective in the discussion of healthy aging is gender. Gender affects individual health status and is closely linked to social roles, resource allocation, and access to healthcare services. As mentioned in a COMMENT article (3) published in *Nature* in May 2024, neglecting sex and gender in research poses a public health risk. In 2020, the WHO released the Decade of Healthy Aging 2020–2030 Action Plan, highlighting that inequities related to healthy aging are particularly prominent in gender disparities, such as widespread poverty among women and gender discrimination. Despite extensive research on aging in China, sex-disaggregated analyses of healthcare utilization, social participation, and resource allocation remain conspicuously absent, hindering the formulation of equitable and inclusive policies.

Under the influence of traditional Chinese culture and social structure, there are significant differences in the aging process between men and women (4). Traditional beliefs dictate that older adult men should shoulder more family and social responsibilities, potentially leading to greater economic pressure during aging (5). Meanwhile, older women generally have lower educational levels, which may hinder their access to health information and self-care (6). Women often assume more caregiving roles in the family and society, potentially compromising their self-care (6, 7). Furthermore, based on China’s urban–rural dual structure, the traditional division of labor in rural households has long-term implications for gender disparities in access to health resources, leading to multiple vulnerabilities for rural older adult women who face a “triple deprivation” characterized by low educational attainment, inadequate social security coverage, and heavy traditional caregiving responsibilities (8). Therefore, a gender-specific exploration of the multifaceted challenges of healthy aging in China not only aids in a more comprehensive understanding of the aging issue but also provides crucial insights for formulating targeted and effective policies. This perspective paper seeks to unravel the multifaceted gender disparities in healthy aging across China’s sociocultural landscape and propose a transformative framework for policy and practice.

## 2 The multidimensional relationship between gender and aging

When delving into the issues of aging, gender is a crucial factor that cannot be overlooked. Men and women face multidimensional differences in the challenges encountered during the aging process, which manifest not only at the physiological level but also in terms of psychological and social adaptation (9).

Throughout the entire lifespan, there are anatomical differences in the brains of men and women. For instance, the male brain exhibits a greater number of neurons and larger volume in specific areas, while the female brain displays more complex structures in the frontal and parietal lobes, providing a biological basis for gender differences in cognitive abilities (10). Men and women also show significant disparities in physiological decline and the incidence of chronic diseases. Elderly men are more susceptible to cardiovascular diseases. Studies have found a stronger association between hypertension and diabetes in older adult men and the incidence of stroke and all-cause mortality compared to women (11). Elderly men have a higher long-term risk of major adverse cardiovascular events following myocardial infarction. As a major cause of cardiovascular diseases, atherosclerosis is influenced by gender as a biological variable. Men exhibit a larger overall plaque burden and higher levels of inflammatory markers compared to women, which can predict cardiovascular events (12).

The more pronounced prevalence of bone-related issues, such as osteoporosis and arthritis, is observed in older adult women. Through a systematic review and meta-analysis of 40 global studies (13) involving 79,127 individuals, it was found that older adult women exhibit a significantly higher prevalence of osteoporosis compared to older adult men (12.5, 95% CI: 9.3–16.7%), reaching 35.3% (95% CI: 27.9–43.4%). This phenomenon is closely associated with the physiological changes that occur post-menopause. As women enter the post-menopausal stage, estrogen levels gradually decline, directly impacting bone health (14). Estrogen plays a critical role in maintaining bone density and strength, and its reduction accelerates bone loss, leading to a significantly increased risk of osteoporosis (15). Furthermore, arthritis is also a common bone-related issue among older adult women, characterized by joint pain, stiffness, and functional impairment, significantly impacting their quality of daily life (16). It is widely acknowledged in academia (17) that post-menopausal osteoporosis is one of the main contributing factors to bone health issues in older adult women.

At the psychological level, older adult men and women have different needs and challenges (18). Men may be more inclined to maintain independence and autonomy, and the decline in physical functioning may lead to a stronger sense of frustration (19). On the other hand, women may place more emphasis on social and emotional support (20, 21). Research has found (22) that compared to older adult men, older adult women are more likely to experience common mental disorders such as depression and anxiety. Therefore, when facing the life changes brought about by aging, women may require more emotional solace and resonance. Interestingly, relevant studies (10, 23, 24) have also found that older adult women tend to be more pessimistic in self-assessing their health, while men, despite being more susceptible to life-threatening illnesses, generally report better health conditions.

Social Determinants of Health (SDH) and cultural factors operate as critical social determinants of health, profoundly shaping the health trajectories of older adult men and women by embedding systemic inequities within societal structures (25). In traditional gender roles, men are often expected to exhibit “strength” and “self-reliance” (26), which may lead them to ignore or self-endure health issues. Conversely, women may be more inclined to seek medical help and social support (27). However, the reality is that when women experience a heart attack, they are more likely to delay seeking assistance, and caregiver interventions are often slower (28). Furthermore, societal stereotypes and biases toward older adults can have negative effects on their self-identity and psychological well-being (29).

## 3 Gender and disease disparities in the older adult population in China

**Figure 1:**
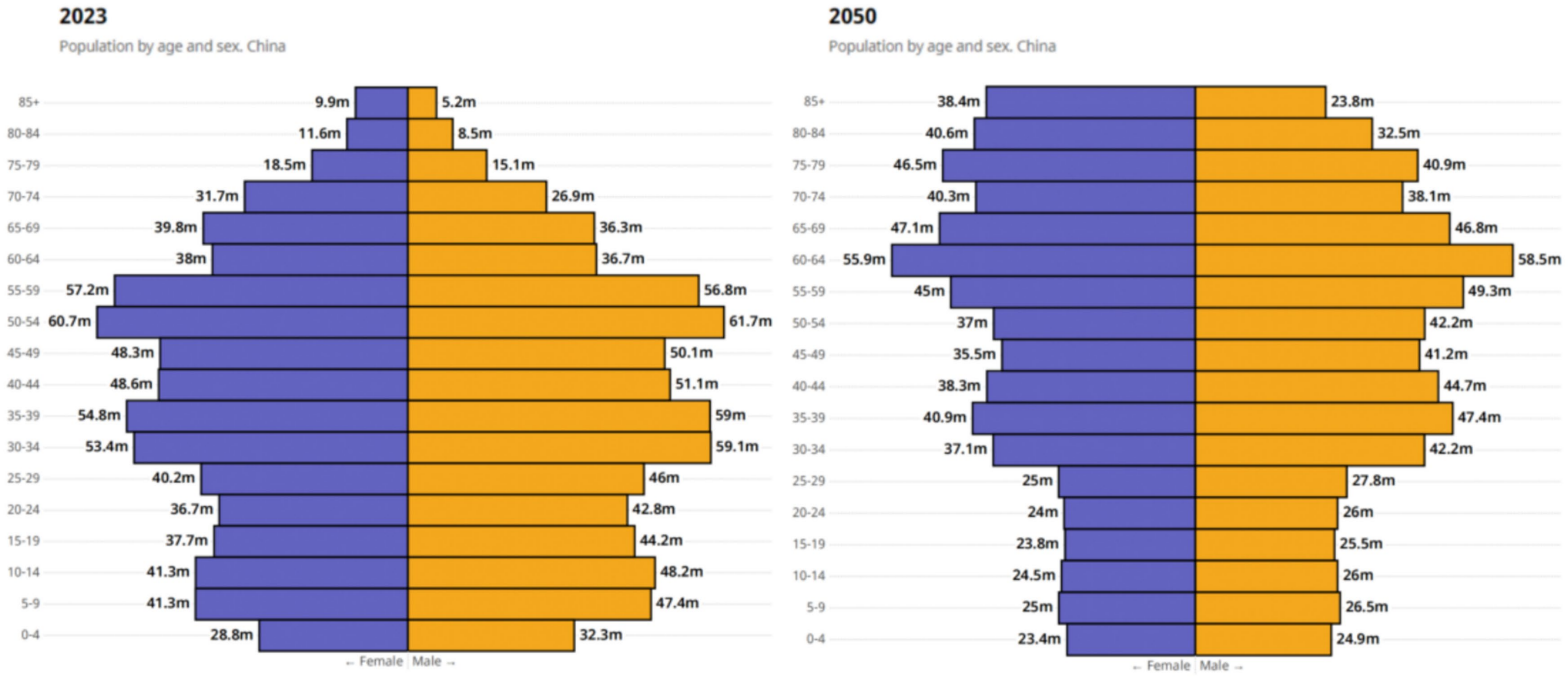
Overview of China’s 2023 population data and 2050 trends by the WHO. Reprinted with permission from WHO, licensed under CC BY-NC-SA 3.0 IGO, https://data.who.int/countries/156 (38).

Note: The image represents the current population situation in China in 2023, while the right image depicts the projected distribution of the Chinese population in 2050 according to the World Health Organization. It is evident from the right image that the population aged 65 and above in China will continue to increase and exhibit an inverted triangle trend. China is facing a significant trend of population aging, with the older adult female population outnumbering the older adult male population as age increases. In the image, “M” represents million.

With the intensifying trend of population aging in China, gender disparities among the older adult population are becoming increasingly prominent. We utilized the GBD 2021 database to analyze the gender differences in population structure for the years 1990, 2000, 2010, and 2020 through the construction of population pyramid graphs (Supplementary material 1). Our analysis revealed that the population structure in 1990 exhibited a typical inverted pyramid shape, with a decreasing proportion of young individuals and an increasing proportion of middle-aged and older adult individuals. Specifically, from 1990 to 2020, the number of individuals aged 80 and above in China experienced a dramatic surge, with the total count skyrocketing from 7.33 million to 31.69 million, representing a staggering three-fold increase. This significant growth not only highlights the rapid acceleration of China’s societal aging trend but also underscores the severity of the aging issue. It is worth noting that, throughout this growth process, the female older adult population consistently occupied a larger proportion. While this is partly attributable to the longer average lifespan of females, it further emphasizes the complexity of gender disparities in aging. In 1990, the female older adult population was nearly 1.7 times higher than the male older adult population (female/male: 4.65/2.68 ≈ 1.74). In 2020, the female older adult population still outnumbered males, with a female-to-male ratio of approximately 1.51 (female/male: 19.07/12.62 ≈ 1.51). Although the gap has slightly narrowed, it demonstrates the advantage of females in terms of longevity. According to the latest statistical data in China, combined with WHO predictions,^3^ the trend of population aging in China is projected to become even more pronounced by 2050 (Figure 1).

According to the 2019 Global Burden of Disease estimation data, China faces a variety of health challenges. Among them, stroke has become the leading cause of death in China, resulting in 142 deaths per 100,000 people. It is closely followed by ischemic heart disease, which causes death in 123 out of 100,000 individuals. Despite improvements in the burden of cardiovascular diseases in China over the past two decades, they still remain one of the primary causes (30). Generally, for the older adult, it is common to have multiple diseases, often coexisting with various chronic conditions. Specifically, research (31) has found that among the 11 widely prevalent diseases, three categories are predominant: cardiovascular diseases (hypertension, stroke, and coronary heart disease), endocrine metabolic diseases (diabetes and metabolic disorders) and musculoskeletal disorders, particularly osteoporosis and osteoarthritis. Therefore, the prevalence of multiple diseases is also a risk factor. A recent study (32) analyzed the prevalence of multiple diseases among middle-aged and older adult individuals (aged ≥45 years) in China, which was found to be 30.4%. This proportion increased to 35.1% when focusing on the population aged 60 years and above. When considering gender differences, the disease prevalence was higher in females compared to males (37.6% vs. 33.4%).

We conducted an in-depth analysis of stroke data in the GBD 2021 database for China (Supplementary material 2), and found that the stroke mortality rate among older adult males was generally higher than that among females across different age groups. Taking the year 1990 as an example, the stroke mortality rate for males in the age group of 65–69 was as high as 868.383583, while for females it was 666.3814602. In the age group of 85 years and above, the stroke mortality rate for males reached 8354.726084, far exceeding the rate for females at 5560.845487. It is worth noting that by the year 2021, although there still remained a gap in stroke mortality rates between males and females, the overall values had decreased, indicating a reduction in stroke mortality rates for both sexes.

Regarding incidence rates, the data from 1990 showed that among the three age groups of 65–79 (65–69; 70–74; and 75–79), males had slightly higher stroke incidence rates compared to females. However, in the age group of 80 years and above, females had slightly higher stroke incidence rates than males. By the year 2021, apart from the age group of 65–69 where males still had slightly higher incidence rates, the differences in incidence rates between males and females were small, with even a slight increase in stroke incidence rates among females compared to males.

From 1990 to 2021, over the span of more than 30 years, both male and female stroke mortality rates have significantly decreased. For example, in the age group of 65–69, the stroke mortality rate for males decreased from 868.38 to 458.65, while for females it decreased from 666.38 to 273.01. This significant change demonstrates the remarkable achievements China has made in the prevention and treatment of stroke (33).

We anticipate further reducing the incidence and mortality rates of stroke through continued efforts and scientific prevention and treatment strategies, bringing health and hope to more patients. At the same time, we need to pay attention to the differences in stroke risk among different age groups and genders, and develop more precise and personalized prevention and treatment approaches.

## 4 Developing gender-specific strategies for healthy aging

Facing the challenges of an aging society in China, it is essential to develop gender-sensitive strategies for healthy aging to accurately meet the health needs of older individuals of different genders. According to the 2020 Seventh National Population Census in China, health conditions were assessed for individuals aged 60 and above (34), with four options provided: healthy, basically healthy, unhealthy but capable of self-care, and unhealthy and incapable of self-care. Looking at the overall situation of the older adult, 57.7% of male respondents reported themselves as “healthy,” compared to 51.9% of females, indicating a higher proportion of males considering themselves healthy. The proportions of males and females reporting to be “basically healthy” were 30.6 and 34.5%, respectively, indicating a higher proportion of females considering themselves basically healthy. Overall, the proportion of healthy males was slightly higher than that of females. According to The Lancet’s report on aging in China published in 2022 (35), Chinese women generally have longer lifespans than men and are more likely to be widowed in later years, which results in them being worse off in almost all dimensions of health compared to men. Furthermore, in terms of health conditions, the gender differences among older adult individuals in China are mainly manifested in the following two aspects:

In the domain of health behaviors, male smoking rates significantly exceed those of females, a habit that potentially elevates the risk of cardiovascular diseases and certain cancers among older adult men. In terms of community care and social interactions, older adult women tend to exhibit lower levels of social participation, particularly following widowhood, leaving them vulnerable to increased social isolation and psychological challenges. This social isolation not only impacts their mental health but also has a negative influence on their overall quality of life. A pivotal finding from a study (27) investigating the incidence of loneliness among older adult individuals in China revealed marked gender disparities. Socioeconomic factors, including education level, occupational choices, and household economic status, exert a more direct influence on loneliness among older adult men. In contrast, older adult women’s sense of loneliness is more profoundly shaped by their self-assessed health status, personal resilience, and the social support they receive. This gender gap underscores the need for targeted measures in addressing loneliness among the older adult, ensuring that both genders receive appropriate care and support.

Currently, we have reviewed a series of policies related to population aging in China and found that while China is actively addressing population aging, strategies specifically targeting the prominent gender differences in aging are currently lacking. Therefore, it is urgent to develop gender-specific health education and preventive measures based on these gender differences. This includes the development and implementation of health education programs tailored to older individuals of different genders, such as interventions targeting male smoking and alcohol consumption, as well as screening and preventive services for female osteoporosis. In terms of mental health, it is important to provide more psychological support and interventions specifically designed for older adult women, while also encouraging and supporting their participation in community activities and social affairs to enhance their social connections and support networks. Furthermore, from a gender perspective in policy development, considering gender differences is crucial when formulating policies related to healthy aging, ensuring that policies can equitably benefit older adult men and women.

## 5 Future prospects and policy recommendations

Gender not only affects the health status of older adults but is also closely linked to social roles, resource allocation, and accessibility to healthcare services. However, current research and policy practices have certain limitations in integrating a gender perspective and fully recognizing the central role of gender factors in healthy aging. Looking ahead, it is necessary to deepen our understanding of the relationship between gender and healthy aging to comprehensively and systematically address the challenges of an aging society. To this end, we propose the following forward-looking recommendations.

The government should increase investment in gender-sensitive research on healthy aging, encouraging scholars and experts to explore in-depth the impact of gender differences on the health of older adults and provide scientific evidence for policy formulation. Promote the collection and analysis of relevant data to gain a more accurate understanding of the health needs of older adults of different genders.

Currently, significant progress has been made in research on aging and health-related fields in China, such as the China Health and Retirement Longitudinal Study (CHARLS) (36), which has conducted large-scale follow-up and health data information collection. Since its inception in 2011, CHARLS has covered approximately 10,000 households and 17,000 individuals in the national baseline survey, with follow-up surveys conducted every 2 to 3 years. Recently, the follow-up data from the fifth wave of CHARLS (2020)^4^ were officially released on November 16, 2023, providing valuable data support for understanding the health status of older adults in China (37). However, despite such progress, there is still a need to further expand the scale of research. Future efforts should focus on the nationalization of family-based older adult care programs in China. This includes not only expanding the scale of research but also emphasizing the standardization and comprehensiveness of health data collection, as well as in-depth and systematic longitudinal studies.

Strengthen the gender sensitivity of healthcare and social service systems through provider training and gender-disaggregated monitoring. The greatest demand of the older adult population is for health-related services. Therefore, healthcare institutions should provide specialized services tailored to older adults of different genders, such as establishing dedicated health check-up programs and providing gender-specific health counseling. At the same time, community organizations should improve social support networks to provide comprehensive support to older adults in areas such as mental health and social interaction. Short-term interventions might include prioritizing community-based screenings and piloting gender-tailored mental health initiatives, while long-term institutional reforms should focus on mandating sex-disaggregated health reporting and embedding gender competency training into medical education curricula. By aligning immediate actions with sustainable systemic changes, these strategies can foster equitable, inclusive care for older adults across genders.

Finally, promoting gender equality awareness in society is crucial to eliminating gender biases and stereotypes toward older adults. We must recognize that gender differences not only exist in later stages of life but may also begin to affect individuals’ health status from early life. Research has found^5^ that comparing the current health status with the physical health status before the age of 16, older adults who had good health before the age of 16 generally rated their current health higher than those who had poor health before the age of 16. Therefore, it is necessary to pay attention to gender differences from early life and develop relevant policies specifically targeting them. Moreover, there is a need for further strengthening prospective research in this field to help us better understand the impact of gender differences on the health of older adults and develop appropriate interventions.

This study has several limitations. First, reliance on secondary data may underrepresent rural and marginalized groups due to sampling biases. Second, the absence of qualitative insights limits our understanding of lived experiences. Third, cross-sectional designs constrain causal inferences. Future research should adopt mixed-methods approaches to capture nuanced gender dynamics and establish longitudinal cohorts to track aging trajectories. Collaborations with grassroots organizations could further amplify the voices of underserved older adult populations.

In conclusion, gender plays a central role in healthy aging. To promote gender equality, improve the overall health and well-being of older adults, and advance social harmony and sustainable development, we need to approach the issue from multiple levels, including research, policy, services, and societal awareness, to collectively build a gender-sensitive society for healthy aging.

## Data Availability

The original contributions presented in the study are included in the article/Supplementary material, further inquiries can be directed to the corresponding authors.

## Footnotes

^1^ https://www.un.org/zh/observances/older-persons-day

^2^ https://www.stats.gov.cn/xxgk/jd/sjjd2020/202105/t20210511_1817280.html

^3^ https://data.who.int/countries/156

^4^ https://charls.charlsdata.com/pages/Data/2020-charls-wave5/zh-CN.html

^5^ http://charls.pku.edu.cn/
